# Characterizing individuals with elevated sweat chloride results in the absence of CFTR variants

**DOI:** 10.1186/s13023-025-04145-w

**Published:** 2025-11-29

**Authors:** Ishmam Bhuiyan, Frank Y. Chou, James M. Roberts, Alessandro Franciosi, Bradley S. Quon

**Affiliations:** 1 Faculty of Medicine, University of British Columbia, Vancouver, BC Canada; 2 Centre for Heart Lung Innovation, University of British Columbia, Vancouver, BC Canada; 3 Division of Respiratory Medicine, Department of Medicine, University of British Columbia, Room 166 - 1081 Burrard Street, Vancouver, BC V6Z 1Y6 Canada; 4 Department of Radiology, University of British Columbia, Vancouver, BC Canada

**Keywords:** Cystic fibrosis, Genetics, Sweat chloride, CFTR, Diagnosis

## Abstract

**Background:**

Cystic fibrosis (CF) is a multi-system disease caused by CFTR dysfunction. Genetic defects in the CFTR protein cause impaired chloride and bicarbonate secretion on the apical surface of epithelial cells throughout the body. Classically, the diagnosis of CF is established based on a clinical presentation suggestive of CF along with two elevated sweat chloride test results (≥ 60 mmol/L) or the presence of two pathogenic disease-causing *CFTR* variants. This study aimed to characterize and compare a subset of patients who present with a CF-like phenotype and elevated sweat chlorides with (‘CF control’) vs. without (‘cases’) disease-causing *CFTR* variants.

**Results:**

Cases were found to have more upper respiratory tract symptoms (sinusitis, nasal polyps, and recurrent sinus infections) compared to CF controls. Furthermore, cases experienced fewer pulmonary exacerbations per year, had less evidence of bronchiectasis, peribronchial thickening, and mucus plugging on CT scan imaging, and fewer organisms identified on sputum microbiology. Compared to CF controls, cases were also noted to have fewer gastrointestinal and genitourinary manifestations of CF.

**Conclusion:**

The clinical features of patients with elevated sweat chlorides in the absence of *CFTR* variants are distinct from patients with *CFTR* variants and comparable sweat chlorides. Further investigation into this subset of patients may elucidate alternative causes for this CF-like phenotype.

## Introduction

Cystic fibrosis (CF) is an autosomal recessive condition that is caused by pathogenic variants in the CF transmembrane conductance regulator (CFTR) gene [1]. The CFTR protein plays an integral role in the secretion of chloride and bicarbonate ions across the apical surface of epithelial cells and regulates sodium reuptake through the epithelial sodium channel [2–4]. Because CFTR maintains appropriate viscosity and acidity of secretions in multiple epithelial lined ducts and lumens, a defect in this protein results in mucus obstruction and end organ damage [3, 5]. In the gastrointestinal tract, CF can manifest as a blockage of the small intestinal lumen (meconium ileus, distal intestinal obstruction syndrome). People with CF (PwCF) also experience recurrent pancreatitis, pancreatic insufficiency, and consequent malabsorption, caused by pancreatic ductal obstruction secondary to the elevated viscosity of pancreatic secretions [6]. Focal biliary cirrhosis [7] and gallstone disease [8] are also commonly recognized complications.

The most significant manifestations of CF, however, occur in the lungs, where the airways demonstrate chronic inflammation and mucus plugging [5]. The volume of periciliary liquid in the upper and lower airways is disrupted in CF, which results in impaired mucociliary clearance [5]; this deficit in mucociliary clearance, a major defense against airway infections, predisposes PwCF to recurrent pulmonary infections. Furthermore, in the years following the initial exposure to bacteria after birth and the ensuing inflammatory response in endobronchial and peribronchial spaces [5, 9], the airways develop bronchiectatic changes [5], characterized by the infiltration of the bronchial wall by inflammatory cells, mucosal wall thickening, airway dilation, and recurrent infections [10].

In diagnosing CF, clinicians assess for a clinical presentation consistent with CF, evidence of in vivo CFTR dysfunction based on elevated sweat chloride testing (≥ 60 mmol/L), and two disease-causing *CFTR* variants [11]. Those with two intermediate sweat chloride concentrations (30–59 mmol/L) and two CF-causing *CFTR* variants can also be diagnosed with CF [11]. However, these criteria for diagnosis do not fully capture the breadth of patients who present with CF-like phenotypes [12]. There exists a subset of patients who present with elevated sweat chlorides (≥ 60 mmol/L) and a CF-like phenotype in the absence of identifiable pathogenic variants in the *CFTR* gene [13, 14]. Because genetic panels and sequencing have evolved in both complexity and number of variants detected in the past several decades [15], it is possible that older studies that have investigated this cohort of patients may have missed *CFTR* genetic variants.

This study aims to characterize individuals presenting with clinical features of CF in conjunction with at least one elevated sweat chloride (≥ 60 mmol/L) but without any *CFTR* variants identified following comprehensive genetic testing. We hypothesized that individuals with elevated sweat chlorides but without any pathogenic *CFTR* variants would manifest with less severe pulmonary disease and fewer extra-pulmonary manifestations, as CFTR dysfunction alone may not be responsible for their symptoms.

## Methods

### Study design

We conducted a retrospective chart review characterizing individuals with ≥ 1 sweat chloride test result diagnostic for CF (≥ 60 mmol/L) but without disease-causing *CFTR* variants on either allele. Individuals were followed at the CF clinics at St. Paul’s Hospital and BC Children’s Hospital in Vancouver, Canada between 1990 and 2022. In order to classify patients as cases or controls, we collected their sweat chloride (in the absence of any CFTR modulator therapy) and genotyping results (standard panel, expanded panel, sequencing, Multiplex Ligation-Dependent Probe Amplification [MLPA] results for deletion/duplication variants; Tables 1 and 2). All sequencing has been performed since 2009 and included all 27 coding exons and flanking intronic regions. Where relevant, we also collected epithelial sodium channel (ENaC) and carbonic anhydrase XII (CA12) sequencing results as alternate causes of an elevated sweat chloride and a CF-like phenotype [16–19]. Cases were defined as individuals who had no identified *CFTR* variants on standard testing and sequencing, and either 2 or more sweat chlorides ≥ 60 mmol/L or an average sweat chloride value over 50 mmol/L (with at least 1 sweat chloride ≥ 60 mmol/L; Table 1). We selected a comparison CF control group that would be considered milder in disease presentation based on their sweat chloride concentrations. Patients who had 2 pathogenic *CFTR* variants with an average sweat chloride value between 60 and 90 mmol/L (and none above 90 mmol/L) were classified as ‘CF controls’ (Table 2). The pathogenicity of *CFTR* variants were determined by cross-referencing CFTR2, a Cystic Fibrosis Foundation-supported database that classifies *CFTR* variants based on their known predilection to cause clinically identifiable disease [20]. Individuals were excluded from this study if they failed to meet the criteria for inclusion in either the case or CF control groups, or if they received a lung transplant during the study period. Given the small size of our case and control groups, the data in this study were analyzed in a descriptive manner, without performing statistical comparisons.

**Table 1:** Summary of cases, including sex, *CFTR* alleles, ethnicity, year of diagnosis, genetic testing performed (and year performed), and sweat chloride results

Case No.	Sex	Allele #1	Allele #2		Ethnicity	Year of Diagnosis	Genetic Testing Performed	Sweat Chloride 1 (mmol/L)	Sweat Chloride 2 (mmol/L)	Sweat Chloride 3 (mmol/L)
1	M	Negative		Negative	White	2010	Standard Panel (39 mutations; 2010), Sequencing (2011), MLPA (2011)	89	95	
2	M	Negative		Negative	South Asian	2000	Sequencing (2009), MLPA (2009)	57	70	
3	F	Negative		Negative	White	2007	Standard Panel (29 mutations; 2007), Sequencing (2010), MLPA (2010), ENaC Sequencing (2014)	89	88	88
4	F	Negative		Negative	White	2017	Expanded panel (139 mutations; 2017), Sequencing (2017), MLPA (2017)	68	73	37
5	F	Negative		Negative	White	2013	Standard Panel (39 mutations; 2014), Sequencing (2015), MLPA (2017)	81	102	
6	M	Negative		Negative	Asian	2011	Sequencing (2012), MLPA (2015)	62	55	
7	F	Negative		Negative	White	2015	Standard Panel (39 mutations; 2013), Sequencing (2015), MLPA (2015)	72	78	
8	F	Negative		Negative	White	2018	Expanded panel (139 mutations; 2017), Sequencing (2018)	71	49	
9	M	Negative		Negative	South Asian	2016	Sequencing (2016), MLPA (2016)	89	54	
10	F	Negative		Negative	White	2016	Standard Panel (39 mutations; 2016), Sequencing (2017), MLPA (2017)	89	77	58
11	F	Negative		Negative	White	2018	Expanded Panel (139 mutations; 2018), Sequencing (2018)	63	75	

**Table 2:** Summary of CF controls, including sex, *CFTR* alleles, ethnicity, year of diagnosis, genetic testing performed (and year performed), and sweat chloride results

CF Control No.	Sex	Allele #1	Allele #2		Number of Disease-causing Variants	Ethnicity	Year of Diagnosis	Genetic Testing Performed	Sweat Chloride 1 (mmol/L)	Sweat Chloride 2 (mmol/L)	Sweat Chloride 3 (mmol/L)
1	M	3849 + 10kbC->T		L218X	2	South Asian	2019	Sequencing (MiSeqDx Cystic Fibrosis Variant Assay, Illumina, Inc.; 2018)	60	72	
2	M	F508del		R1066H	2	White	1984	Standard Panel (27 exons, part of intron 19, part of intron 8; 1998)	71		
3	M	F508del		G85E	2	White	2000	Standard Panel (12 mutations; 2000),	86		
4	M	3849 + 10KbC > T		Exon 2-3del	2	White	2002	Standard Panel (20 mutations; 2002), Sequencing (2015), MLPA (2015)	85		
5	M	F508del		R117H − 5T	2	White	2002	Standard Panel (29 mutations; 2003)	70	73	56
6	F	F508del		F508del	2	White	1989	Unknown	83		
7	F	F508del		F508del	2	First Nation	1992	Unknown	70	90	
8	M	F508del		L206W	2	White	2014	Standard Pnael (39 mutations; 2013), Sequencing (2014), MLPA (2014)	70	68	
9	M	F508del		F508del	2	White	2000	Standard Panel (12 mutations; 2000),	77	85	
10	F	N1303K		S489X	2	White	1997	Standard Panael (12 mutations; 1997), Sequencing (2011), MLPA (2011)	90		

### Variables

Paper clinical charts served as our primary data source, and electronic medical records were used as a supplement for data that were not available in the paper charts. Patients’ current age, sex, age at diagnosis, symptoms at diagnosis (upper respiratory, lower respiratory, gastrointestinal, genitourinary), family history of CF, and smoking history (tobacco and cannabis) were included. Where relevant, we also recorded individuals’ age and cause of death. To characterize the clinical features of the individuals included in our study, we determined whether they had the following CF-related comorbidities at any time during follow up: concomitant asthma, sinusitis, gastroesophageal reflux disease (GERD), delayed gastric emptying, liver disease, bowel obstruction or constipation, pancreatic insufficiency, pancreatitis, diabetes, infertility, low bone mineral density, and fat-soluble vitamin deficiency. We also included sputum microbiology before and after diagnosis, the number of pulmonary exacerbations requiring IV or PO antibiotics per year during the study period, and whether patients were on pancreatic enzyme replacement therapy or not.

As the age of diagnosis of cases (median = 41, mean = 42) was older than the CF controls (median = 7, mean = 10.7), we analyzed clinical characteristics closest to the date of diagnosis for cases and the most recent available results for CF controls to optimize our comparison of clinical features at a similar age.

### CT scoring

To compare commonly observed features of CF on lung imaging, CT chest scans were reviewed and scored according to the Brody scoring system [21, 22], which assigns a global score for bronchiectasis, mucus plugging, peribronchial thickening, parenchymal involvement, and focal air trapping. In an email from Dr. Alan Brody in February 2019, detailed instructions on using this scoring system were shared and subsequently used in this study (Dr. Alan Brody, Email, 2019 February 11). The Brody scoring system uses inspiratory images for each observation other than air trapping and assigns a score for each of the 6 lung lobes, including the lingula. Each lobe was given a score of 1 for each feature if it is present in less 33% of the lobe, 2 if it is seen in 33–67% of the lobe, and 3 if it is noted in more than 67% of the lobe (by volume). Airway size and wall thickness were used to assess the severity of peribronchial thickening and bronchiectasis. The largest and average size of dilated bronchi were scored based on their size in relation to the accompanying vessel (< 2x = 1; 2x-3x = 2; >3x = 3). Furthermore, the severity of airway wall thickening was scored based on its proportion in relation to the diameter of the blood vessel. Each lobe was scored separately and a maximum score of 36.5 was possible for each lobe. The score for each CT abnormality was also averaged to create a global score across all of the 6 lung lobes.

## Results

### Baseline clinical characteristics

A total of 21 individuals were included in this study, of whom 11 were classified as cases (Table 1) and 10 were classified as CF controls (Table 2). 4 of the cases (36%) and 7 (70%) of the CF controls were males. Both the cases (median = 75.5; IQR = 15.5) and CF controls (median = 71.5; IQR = 23.9) had comparable average sweat chloride results. Compared to the CF controls (30%), fewer (9%) cases had a family history of cystic fibrosis, and a higher proportion of cases (18%) than CF controls (10%) had a smoking history. A comparable proportion of cases (36%) and CF controls (30%) had concomitant asthma. Although we did not systematically test individuals in this study for epithelial sodium channel (ENaC) carbonic anhydrase XII (CA12) mutations, one of the cases underwent epithelial sodium channel (ENaC) sequencing, yielding negative results.

### Signs and symptoms at diagnosis

Signs and symptoms at diagnosis were compared between the two groups. Both gastrointestinal and genitourinary manifestations, the latter which included congenital absence of the vas deferens or azoospermia on semen analysis, were noted more frequently in the CF controls than cases (Table 3**)**. Individuals from the case group more frequently had sinonasal symptoms (due to chronic sinusitis or nasal polyps) or a history of sinus infection compared to CF controls. When grouped together, upper respiratory tract symptoms—such as sinusitis, nasal polyps, and recurrent sinus infections—were noted in 64% of cases and 33% of CF controls. Lower respiratory tract symptoms, which included recurrent chest infections, chronic cough, and sputum production, were noted in 64% of cases and 78% of positive controls. While the median FEV1 values were comparable between cases (87% predicted, 2.9 L) and CF controls (79% predicted, 3.1 L), the median BMI of the cases (19.7 kg/m^2^) was lower than the CF controls (23.5 kg/m^2^). Cases were also less likely to demonstrate sputum positivity for various microorganisms relative to their CF controls (Table 3).

**Table 3:** Summary of clinical variables, including demographic data, symptoms at diagnosis, spirometry, BMI, sputum microbiology, CF comorbidities, laboratory results, and CT bronchiectasis scores of CF controls vs. cases. Abbreviations: Q1 = first quartile; Q3 = third quartile; CT = computed tomography; ENT = ear, nose, throat; FEV1 = forced expiratory volume in 1 s; BMI = body mass index; MRSA = methicillin-resistant Staphylococcus aureus; GERD = gastroesophageal reflex; PERT = pancreatic enzyme replacement therapy; SD = standard deviation

Category	Variable	Cases (*n* = 11)	CF Controls (*n* = 10)	
**Demographic Data**	Male (%)	4 (36.4%)	7 (70.0%)	
	Current Age: Median (Q1, Q3)	47.5 (37.8, 63.0)*	32.0 (25.3. 37.5)	
	Age at Diagnosis: Median (Q1, Q3)	41.0 (24.0, 61.5)	7.0 (0.0, 17.5)	
	Sweat Chloride: Median (Q1, Q3)	71.5 (61.8, 85.7)	75.5 (67.0, 82.5)	
	Family History of Cystic Fibrosis (%)	1/11 (9.1%)	3/10 (30.0%)	
	Smoking History (%)	2/11 (18.2%)	1/10 (10.0%)	
	Concomitant Asthma (%)	4/11 (36.4%)	3/10 (30.0%)	
**Signs and Symptoms at Diagnosis**	Sinusitis (%)	7/11 (63.6%)	3/9 (33.3%)	
	Nasal Polyps (%)	2/11 (18.2%)	1/9 (11.1%)	
	Recurrent Sinus Infections (%)	1/11 (9.1%)	0/9 (0.0%)	
	All Upper Respiratory (%)	7/11 (63.6%)	3/9 (33.3%)	
	Recurrent Chest Infections (%)	4/11 (36.4%)	4/9 (44.4%)	
	Chronic Cough (%)	2/11 (18.2%)	3/9 (33.3%)	
	Sputum Production (%)	1/11 (9.1%)	2/9 (22.2%)	
	All Lower Respiratory (%)	7/11 (63.6%)	7/9 (77.8%)	
	Gastrointestinal (%)	3/11 (27.3%)	6/9 (66.7%)	
	Genitourinary (%)	1/11 (9.1%)	2/9 (22.2%)	
**Spirometry**,** BMI**	FEV1 (%): Median (Q1, Q3)	87.0 (75.5, 97.5)	78.5 (65.0, 92.5)	
	FEV1 (L): Median (Q1, Q3)	2.9 (1.9, 3.7)	3.1 (2.2, 4.0)	
	BMI: Median (Q1, Q3)	19.7 (17.8, 25.1)	23.5, 21.9, 28.6)	
**Sputum Microbiology (Prior to Diagnosis)**	*Pseudomonas aeruginosa* (%)	1/11 (9.1%)	0/10 (0.0%)	
	*Staphylococcus aureus* (%)	1/11 (9.1%)	2/10 (20.0%)	
	*Haemophilus influenzae* (%)	0/11 (0%)	1/10 (10.0%)	
	Mycobacterium Avium Complex (%)	0/11 (0%)	0/10 (0.0%)	
**Sputum Microbiology (Post Diagnosis)**	*Pseudomonas aeruginosa* (%)	0/11 (0%)	4/10 (40.0%)	
	*Staphylococcus aureus* (%)	5/11 (45.5%)	9/10 (90.0%)	
	MRSA (%)	0/11 (0%)	2/10 (20.0%)	
	Haemophilus influenzae (%)	0/11 (0%)	3/10 (30.0%)	
	Stenotrophomonas (%)	1/11 (9.1%)	3/10 (30.0%)	
	Aspergillus (%)	0/11 (0%)	6/10 (60.0%)	
	Mycobacterium Avium Complex (%)	1/11 (9.1%)	1/10 (10.0%)	
**Number of Pulmonary Exacerbations During Study Period**	Number of Pulmonary Exacerbations Per Year During Study Period: Median (Q1, Q3)	2.9 (0.7, 9.6)	12.1 (4.3, 22.7)	
**CF Comorbidities**	GERD (%)	6/11 (54.5%)	7/10 (70.0%)	
	Delayed Gastric Emptying (%)	1/11 (9.1%)	2/10 (20.0%)	
	Liver Disease (%)	1/11 (9.1%)	2/10 (20.0%)	
	Bowel Obstruction (%)	1/11 (9.1%)	0/10 (0%)	
	Pancreatic Insufficiency (on PERT and/or fecal elastase < 200 ug/g stool) (%)	3/11 (27.3%)	5/10 (50.0%)	
	Pancreatitis (%)	1/11 (9.1%)	1/10 (10.0%)	
	Diabetes (%)	1/11 (9.1%)	4/10 (40.0%)	
	Infertility (% of Male Patients)^^^	1/4 (25.0%)	2/7 (28.6%)	
**CT Bronchiectasis** **(Global scores)**	Bronchiectasis: Mean (SD)	5.2 (5.0)*	14.2 (12.5)**	
	Mucus: Mean (SD)	4.1 (3.3)*	9.8 (6.3)**	
	Thickening: Mean (SD)	3.9 (2.0)*	13.4 (11.7)**	
	Parenchyma: Mean (SD)	1.2 (1.8)*	1.2 (1.3)**	
	Air Trapping: Mean (SD)	0.0 (0)*	0.0 (0)**	

* Data available for 10/11 patients

** Data available for 9/10 patients

^ Infertility defined based on CBAVD or absence of sperm on semen analysis

### CF comorbidities and complications

Fewer individuals classified as cases had gastrointestinal manifestations of CF, such as pancreatic insufficency and liver disease (Table 3). Fewer cases were prescribed pancreatic enzyme replacement therapy (PERT; 27%) and had fecal elastase measurements below 200 micrograms/g stool (0%) compared to CF controls (50% on PERT; 10% with fecal elastase below 200 micrograms/g stool). A similar proportion of cases (9%) and CF controls (10%) were reported to have a history of pancreatitis. A lower proportion of cases had diabetes (9%) compared to CF controls (40%). Cases had a lower median number of pulmonary exacerbations per year (2.9) than CF controls (12.1).

### CT findings

Based on the Brody scoring system, cases had a lower mean score for bronchiectasis (5.2 vs. 14.2), mucus plugging (4.1vs. 9.8), and peribronchial thickening (3.9 vs. 13.4) compared to CF controls, while both groups had similar scores for parenchymal involvement (Table 3).

## Discussion

In this study, we characterized individuals with elevated sweat chlorides in the absence of *CFTR* variants and compared them to individuals with comparable sweat chloride results with at least 1 CF-causing variant. We found that our cases had lower BMI, higher FEV1 (%), less positivity for typical CF pathogens on sputum microbiology and less evidence of bronchiectasis, mucus plugging, and peribronchial thickening on CT imaging when compared to CF controls. However, our cases had more upper respiratory tract symptoms, such as nasal polyps, sinusitis, and recurrent sinus infections. We also found that our cases tended to have fewer gastrointestinal and genitourinary manifestations compared to CF controls.

Few studies have described the phenotype of individuals with elevated sweat chlorides (≥ 60 mmol/L) but without *CFTR* variants identified following clinically available genetic analysis. Mekus et al. [14] published a case report of a patient who presented with clinical features of CF without identifiable *CFTR* variants, including recurrent bronchitis, excessive sputum production, chronic cough, a history of pneumonia, and elevated sweat chlorides. No *CFTR* variants were found when sequencing the 27 exons and flanking intronic sequences [14]. In another study by Groman et al. [13], extensive genetic analysis of the *CFTR* gene including DNA sequencing of the 27 exons, flanking intronic sequences, and 2 intronic variants known to cause abnormal splicing was performed on 74 patients with “non-classic” CF clinical presentations defined by two or three of the following manifestations of CF: elevated sweat chloride >40 mmol/L, CF lung disease, CF-related gastrointestinal disease, or malformation of the vas deferens. Thirty of 74 (41%) individuals did not have any detectable *CFTR* variants following DNA sequencing, including additional genetic analysis evaluating for genomic rearrangements and sequencing of the basal promoter regions. Individuals from this study with 0, 1 or 2 *CFTR* variants were clinically indistinguishable in terms of multi-organ involvement.

The diagnostic classification of individuals with sweat chloride ≥ 60 mmol/L but no identifiable CFTR variants can be challenging to reconcile for CF clinicians because of the discordant results. Under current CF diagnostic guidelines [11], all of the cases in our cohort meet criteria for CF based on elevated sweat chloride values alone, independent of *CFTR* genotyping. Nonetheless, some individuals may be reclassified as having a CFTR-related disorder [23] or as non-CF following more extensive functional and genetic CFTR evaluation. Evidence of CFTR dysfunction from in vivo nasal potential difference (NPD) and intestinal current measurement (ICM) testing [24, 25], or from ex vivo assays using patient-derived intestinal or airway cells (e.g., forskolin-induced organoid swelling) [26, 27], would strengthen diagnostic confidence; however, access to these tests remain limited to specialized centres because of the technical expertise required [28, 29].

A limitation of our study—and of prior reports—is that *CFTR* genetic analysis has relied primarily on sequencing of the coding region of *CFTR* with limited intronic coverage, along with multiple ligation-dependent probe amplification (MLPA) to detect copy number variants (e.g. deletions/duplications), collectively referred to as ‘conventional genetic testing’. Expanded CFTR molecular testing—including next generation sequencing (NGS)-based copy number variant analysis, deep intronic/regulatory sequencing, long-range and/or targeted NGS or whole-genome sequencing to detect splice-altering intronic variants, polyT/TG tract genotyping of intron 8 (e.g., 5T / TG11-13), and RNA (cDNA) studies from patient-derived epithelial cells to reveal aberrant splicing—are not widely available but should be considered to detect additional *CFTR* variants [30–33]. There is recent evidence suggesting that deep intronic sequencing can identify additional *CFTR* variants, but the yield depends on the number of variants detected by initial comprehensive *CFTR* sequencing. Through whole-gene sequencing including non-coding regions of *CFTR*, Sheridan et al. [34] identified a second variant in 47% of patients in the CF Variant Analysis Program (MAP) and 45% of patients in the CF Genome Project (CGFP) who had one previously identified *CFTR* variant. However, identifying a second variant occurred rarely or not at all in those with no identified variants (7% among MAP patients and 0% among CFGP patients). Taken together, these findings suggest that individuals with a CF-like phenotype, sweat chloride >60 mmol/L, and only one identified *CFTR* variant (following conventional genetic testing) may benefit from additional intronic sequencing. However, for individuals with no *CFTR* variants identified following conventional genetic testing, as was the case in our study, intronic sequencing is unlikely to have additional diagnostic yield; accordingly, the likelihood of false-negative genetic test result is low.

When elevated sweat chloride is observed without detectable disease-causing *CFTR* variants, alternative explanations should be considered. Guglani et al. (2015) catalogued false-positive sweat chloride results across 11 categories: renal, dermatologic, endocrine, genetic, nutritional, neurologic, medications, psychosocial, toxic (e.g. arsenic poisoning), immune, and gastrointestinal [35]. Many listed conditions—e.g., untreated Addison disease, hypoparathyroidism, and nephrogenic diabetes insipidus—do not present with a CF-like phenotype; reported associations with elevated sweat chloride were often incidental or uncovered during evaluation for another condition [35]. Because most of the individuals in our cohort exhibited CF-like manifestations, these non-CF causes for a positive sweat chloride test are less likely; rather, these cases are more likely to represent CF (or a CFTR-related disorder) despite negative clinical genotyping.

Alternatively, variants in other non-*CFTR* genes, namely the beta-unit of the epithelial sodium channel (ENaC) and carbonic anhydrase XII (CA12) have been reported to be associated with elevated sweat chlorides and CF-like clinical presentations [16–19]. Sheridan et al. [18] described patients with deleterious SCNN1B as having recurrent pulmonary infections and elevated sweat chlorides in the absence of renal manifestations. Lee et al. [17] described a proband of two pedigrees with CA12 variants who developed bronchiectasis and elevated sweat chlorides, but no notable gastrointestinal manifestations of CF, other than self-resolving pancreatic insufficiency. Like these previous studies that focused on ENaC and CA12, our study also found that patients without *CFTR* variants tended to have more isolated airway manifestations, highlighting the need to consider whole genome sequencing to identify variants in these non-CFTR genes.

In addition to non-CF genetic causes, there is emerging evidence that environmental factors can also contribute to a CF-like phenotype. Acrolein in cigarette smoke causes acquired CFTR dysfunction, even in people without genetic variants in the *CFTR* gene. In addition to causing direct CFTR dysfunction in the airways, there is evidence of systemic extra-pulmonary effects [36–38], including reduced intestinal CFTR function [39] and elevated sweat chlorides [39]. However, in our study, higher smoking rates were not found among cases when compared to CF controls, suggesting against this explanation in the group of patients we studied.

There are several limitations of this study. Firstly, the sample size was limited as can be expected when studying a rare subset of patients. Secondly, due to limitations in access to clinical genetic testing, we did not systematically evaluate patients for the presence of ENaC or CA12 variants, both of which are potential causes of elevated sweat chlorides and CF-like disease in patients without disease-causing *CFTR* variants. Thirdly, as a result of the rarity of this subset of patients, it was difficult to find a CF control group that matched our identified cases in terms of age, sex, and sweat chloride results. In our CF control group, which included milder CF individuals with sweat chlorides just above the diagnostic threshold, we had over-representation of males likely due to the diagnosis of CF during infertility work-up, many of which have fewer clinical manifestations of CF as the vas deferens is the most sensitive to CFTR dysfunction. Fourthly, analytical error in sweat chloride testing could have affected our data as deviations in sweat chlorides of up to 41% can be attributed to technical factors such as pipetting errors [40]. This, again, highlights the important of using a variety of CFTR functional and genetic tests to approach cases of CF-like disease in the absence of disease-causing *CFTR* variants.

In conclusion, individuals with a CF-like phenotype and elevated sweat chlorides without *CFTR* variants identified tend to have lower BMI, more upper respiratory tract symptoms at the time of diagnosis, and fewer gastrointestinal and genitourinary manifestations classically associated with CF. More extensive functional and genetic evaluation of CFTR should be considered to support/refute a diagnosis of CF in these discordant cases along with an investigation into alternative causes of a CF-like phenotype reported in the literature including variants in genes other than *CFTR*, such as the beta-unit of ENaC and CA12.

## Data Availability

The datasets used and/or analyzed during the current study are available from the corresponding author on reasonable request. Relevant figures and tables are included in this published article.

## Abbreviations

CF: Cystic fibrosis
(CFTR) gene: Cystic fibrosis transmembrane conductance regulator
MLPA: Multiplex Ligation-Dependent Probe Amplification
ENaC: Epithelial sodium channel
CA12: Carbonic anhydrase XII
ENT: Ear-nose-throat
